# Endovascular retrieval of a dislocated coil in the peroneal artery with a stent retriever

**DOI:** 10.1259/bjrcr.20150278

**Published:** 2016-01-19

**Authors:** Omid Nikoubashman, Rico Badenschier, Marguerite Müller, Carolin Brockmann, Gerrit Schubert, Marc-Alexander Brockmann, Georg Mühlenbruch, Hans Rainer Clusmann, Martin Wiesmann

**Affiliations:** ^1^ Department of Neuroradiology, University Hospital Aachen, Aachen, Germany; ^2^ Institute of Neuroscience and Medicine 4, Forschungszentrum Jülich GmbH, Jülich, Germany; ^3^ Department of Radiology, Helios Klinikum Schwerin, Schwerin, Germany; ^4^ Department of Neurosurgery, University Hospital Aachen, Aachen, Germany; ^5^ Department of Radiology, Medizinisches Zentrum StädteRegion Aachen GmbH, Würselen, Germany

## Abstract

We present a patient who underwent successful removal of a fully detached platinum coil from the peroneal artery using a Solitaire^™^ stent retriever (Covidien, Irvine, CA) that is usually used in endovascular stroke treatment.

## Summary

We present a patient whom underwent successful removal of a fully detached platinum coil from the peroneal artery using a Solitaire^™^ stent retriever (Covidien, Irvine, CA) that is usually used in endovascular stroke treatment.

## Introduction

Endovascular coil embolization has become a common treatment option for arterial aneurysms. Nevertheless, coiling of wide-neck, giant or very small aneurysms can be challenging and therefore sometimes be associated with complications. Coil misplacement and dislocation have been reported to occur in up to 6% of procedures during endovascular aneurysm treatment and can cause ischaemic complications if left untreated.^1–3^ Complication management has always received little attention and although it can be necessary to remove a fully detached coil in some cases, there is no standard rescue procedure for this problem^3^. The effective use of stent retrievers, which are usually used in endovascular stroke treatment and therefore available in a large number of endovascular centres, was systematically investigated in an animal model but has so far been described in only a few patients.^3–7^ We present a case in which a fully detached coil was extracted from a peripheral artery using a Solitaire stent retriever that was primarily designed for thrombectomy in acute stroke treatment.

## Case Report

**Figure 1.**
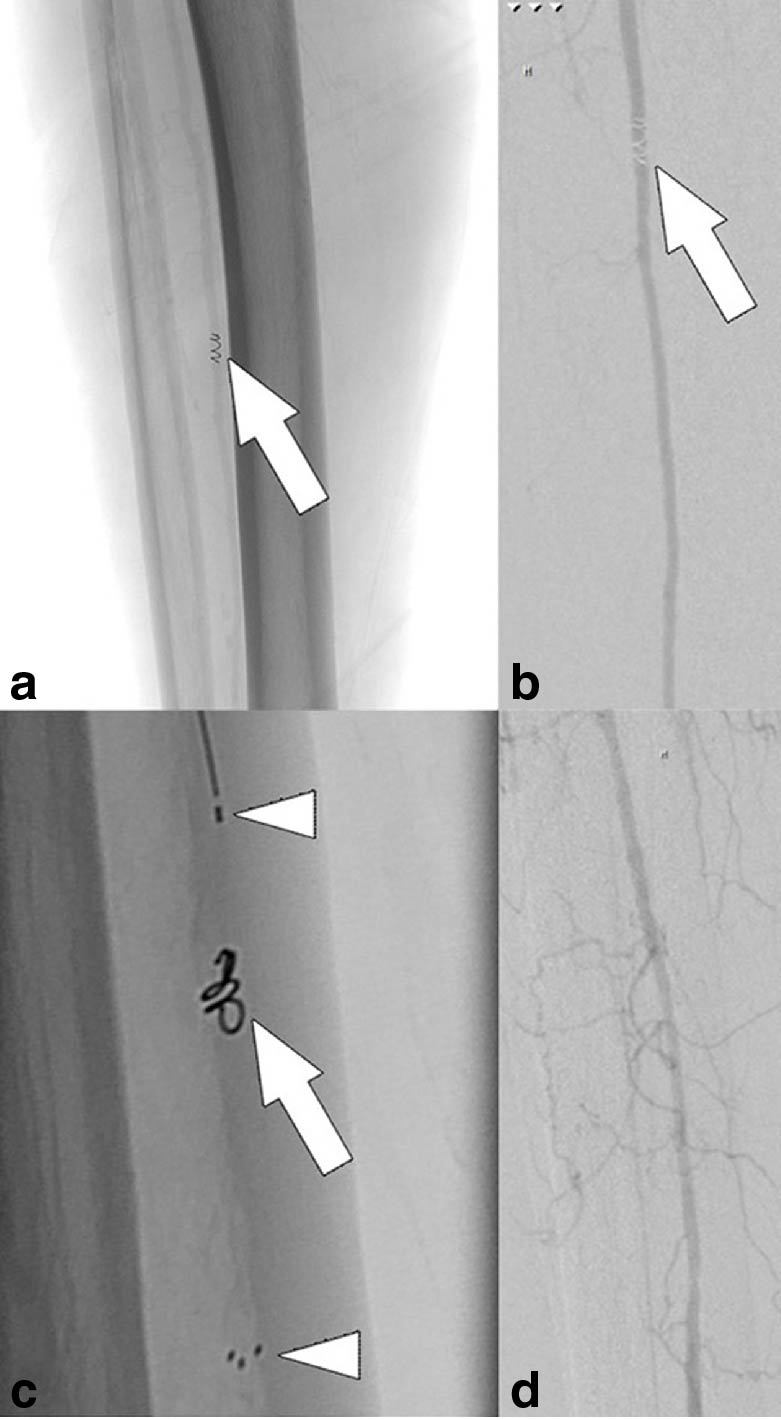
A DSA showing a dislocated coil (VortX^™^ Diamond 3 × 3.3 mm) in the right peroneal artery (a–c; arrows). The coil caused an occlusion of the peroneal artery and subsequent hypoperfusion of the respective muscle branches (b). A Solitaire^™^ stent retriever (4 × 20 mm; Covidien, Irvine, CA) was placed in the peroneal artery covering the dislocated coil (c; upper and lower arrowheads indicating the proximal and the distal marker of the stent, respectively). The coil was then locked within the stent mesh by pushing the microcatheter and pulling the stent retriever. The coil was then carefully recovered by pulling both the stent retriever and the microcatheter through the inguinal sheath. DSA documents regular flow after coil retrieval (d). DSA, digital subtraction angiography.

An elderly male patient suffering from severe epistaxis was treated with coil-assisted particle embolization of the sphenopalatine artery. During embolization a malfunctioning coil (VortX^™^ Diamond 3 × 3.3 mm, Boston Scientific, Marlborough, MA) was stuck at the tip of the coiling microcatheter (Rebar 18^™^, Covidien, Mansfield, MA) and could not be recovered. A decision was made to remove the coil by retrieving the microcatheter through a short 6 French inguinal sheath (Cordis, Miami Lakes, FL). For this purpose, the guiding catheter (6 French Envoy MPD with an angled tip, Cordis) was removed first. However, the coil became unhitched and was lost when the microcatheter was being pulled through the sheath. The coil migrated into the peroneal artery, which was the patient’s main supplying artery of the lower limb (Figure 1a). The coil caused hypoperfusion of the subsequent muscle branches (Figure 1b). Consequently, the decision was made to retrieve the detached coil with a Solitaire stent retriever. A rescue attempt with dedicated retrieval devices such as the “snare” or “lasso” device or the Alligator^®^ device (Chestnut Medical Technologies, Menlo Park, CA) was not made, as the interventionalist lacked sufficient experience with these devices.

For this procedure, the interventionalist introduced a short anterograde sheath (6 French, Cordis), through which a Rebar 18 microcatheter was pushed through the lost coil. A Solitaire stent retriever (4 × 20 mm) was introduced through the microcatheter and opened in the peroneal artery covering the dislocated coil (Figure 1c). The coil was locked within the stent mesh by carefully pushing the microcatheter while pulling the stent retriever. The coil was then carefully retrieved by pulling back the stent retriever together with the microcatheter into the inguinal sheath.

A control series showed normal flow (Figure 1d) and no perforation or thrombosis of the peroneal artery. The patient was symptom free after the procedure and remained so during a follow-up of 18 months.

## Discussion

Misplacement or dislocation of coils during aneurysm embolization is reported to occur in 3–6% of procedures.^1,2^ When detached coils pose a risk for subsequent vessel occlusion, it may be necessary to retrieve the whole coil.^3^ Coil retrieval with devices that are dedicated for retrieval of foreign material, such as the Alligator or the lasso device, has been reported to be effective in approximately 81% (29/36) of cases.^7,8^ Although no complications have been reported, coil retrieval with the previously mentioned devices is reported to be more challenging and requires more manipulation than coil retrieval with stent retrievers.^3^ Given the relatively stiff tip of the Alligator device, access to distal or curved vessels with this device can be particularly challenging.^3^ Recovery of fully detached coils with stent retrievers, however, has been reported to be highly effective and less challenging.^3–7^ Success rates and optimal handling have been investigated in an animal model, where 101 of 102 retrieval manoeuvres were successful, regardless of coil type, size and shape when the coil was locked within the stent mesh by advancing the microcatheter over the stent.^7^ Coil retrieval in patients has been reported with various stent retrievers, namely the Solitaire, the Trevo^™^ (Stryker, Kalamazoo, MI) and the Catch device (Balt, Montmorency, France), and was successful in all 19 reported cases.^3–6,8^ While all published cases were dealt with intracranial coils, we also showed that it is possible to recover a small coil in a peripheral artery with a small diameter. In summary, coil retrieval with stent retrievers can be considered an effective treatment option in a vast variety of settings.

## Learning points

1. Misplacement or dislocation of coils during aneurysm embolization is reported to occur in 3–6% of procedures.^1,2^
2. Recovery of fully detached coils with stent retrievers is effective if performed correctly.^3–7^
3. When utilizing a stent retriever to recover a coil, the coil can be locked within the stent retriever by pushing the microcatheter and slightly pulling the stent retriever.^3,4,7^

## Consent

Informed consent was obtained from all individual participants included in this study.
